# Reply to the January Editorial

**Published:** 1896-03

**Authors:** J. Leon Williams

**Affiliations:** 30 George Street, Hanover Square, London, W.


### Foreign Correspondence.

### REPLY TO THE JANUARY EDITORIAL.

To the Editor:

Sir,—I have just read the strictures on Dr. Black's recent investigations which you contribute in an editorial in the January number of your magazine. I am glad to see the discussion of this most important subject opened by one who, like yourself, has decided opinions. Although I disagree almost *in toto* with your conclusions, yet your point of view leads you to seize upon the most significant aspects of Dr. Black's work, and thus to set clearly before your readers the points at issue. During the past two years I have been engaged in investigations in this same field of research, and although my work has been entirely confined to such methods as may be conducted by the aid of the microscope, I find that my conclusions, as far as reached at present, are largely in harmony with those of Dr. Black. And it appears to me, Mr. Editor, that while the facts which you state are above reproach, as, for example, that some teeth decay much more rapidly than others, and that metal fillings are not always the best for children's teeth, yet your reasoning upon these facts is extremely lame. Whatever direction our theories may take with reference to caries of the teeth, we have to keep them within the broad lines of facts as known. Now, I consider it a firmly-established fact that slowness or rapidity of decay of the teeth is not primarily dependent upon the exact amount of lime salts which they may contain. Foremost in the evidence in support of this position must be placed the facts, reached both by microscopic investigations and by analyses, that normal mature enamel[1] contains *no appreciable* per cent. of true organic

[1] By "normal enamel" I do not mean what might be called *average* in liability to decay, but normal in structure from the stand-point of the histologist.

matter. This was practically the position taken up by Drs. Black and Sudduth and myself during the discussion of the paper which I read before the First District Dental Society of New York, December 9, 1885. My recent investigations have confirmed this view, and it has been still more completely established by a recent analysis of enamel made by Mr. Charles Tomes. From my knowledge of the methods employed by Mr. Tomes, I believe the analysis to have been the most careful and accurate ever made. In twenty grains of enamel he found no sufficient quantity of organic matter to weigh, probably, less than 0.1 per cent.,—and even this small quantity might be entirely extraneous. If there be *no* organic matter in enamel, then there can be no question of greater or less liability to caries because of a greater or less quantity of that which does not exist.

But this is only one line of argument in support of our position. When we come to examine the enamel of the teeth of some mammals and fishes we find a more or less elaborate organic structure in it. In marsupial teeth, for instance, we find the dentinal fibres everywhere continued into and distributed throughout the enamel. In the teeth of many fishes the enamel is penetrated by large canals containing organic matter. But these teeth rarely or never decay.

The case, therefore, stands thus: Human teeth, which contain little or no organic matter, often decay rapidly. The teeth of many animals and fishes, containing a large proportion of organic matter, rarely or never decay. To what conclusion are we forced in the face of these facts? Manifestly that the presence or non-presence of organic matter is an unimportant factor in the phenomena of decay of the teeth. The investigations of Drs. Miller and Black on this subject must be ranked as the most important ever made. I hope to supplement this work in a forthcoming series of papers on the results of microscopic investigation. The facts are all on one side of this question. The liability of human teeth to caries is largely due to environing conditions and not to inherent structure. The problem might with equal accuracy be stated in another form by saying that no tooth will decay if its environment be healthy, and no tooth can resist decay if its environment be unhealthy. By "unhealthy" I mean, of course, such particular external conditions as are inimical to the preservation of the tooth. Dr. Miller has shown what these conditions are. It would be going too far to assert that tooth-structure has *no* relation to caries, but even such a statement would be much nearer the truth than the position occupied to-day by a large majority of the dental profession. The judicial mind which can rightly estimate the value of evidence by giving to each and every fact its proper place and importance is not a common possession, and it often takes a long time to turn men's minds from habitual ways of thinking, no matter how absurd the position occupied may be.

One further series of investigations is needed, and the results of this would, I think, give us all the necessary data for a thoroughly scientific formulation of the phenomena of decay of the teeth. Carefully conducted experiments should be made along the lines followed by Dr. Miller for the purpose of determining the relative rapidity of decay of teeth of so-called dense structure as compared with those generally regarded as less resistant to decay. When this work is accomplished we shall, I believe, find the difference far less than is popularly supposed.

It has been estimated that from five to seven years is required for a change in the molecular structure of bone. Dentine changes much more slowly, and nothing is more certainly established than the fact that there is *no* proper nutritive change in human enamel. How illogical, then, the contention that the rapid increase or decrease in decay of the teeth in any particular case is due to change of tooth-structure.

I am, dear sir, yours faithfully,

J. Leon Williams.

30 George Street, Hanover Square, London, W.

